# Image Quality Ranking Method for Microscopy

**DOI:** 10.1038/srep28962

**Published:** 2016-07-01

**Authors:** Sami Koho, Elnaz Fazeli, John E. Eriksson, Pekka E. Hänninen

**Affiliations:** 1 Laboratory of Biophysics, Department of Cell Biology and Anatomy, Institute of Biomedicine and Medicity Research Laboratories, University of Turku, Turku, Finland; 2 Cell Biology, Biosciences, Faculty of Science and Engineering, Åbo Akademi University, Turku, Finland; 3 Turku Centre for Biotechnology, University of Turku and Åbo Akademi University, Turku, Finland

## Abstract

Automated analysis of microscope images is necessitated by the increased need for high-resolution follow up of events in time. Manually finding the right images to be analyzed, or eliminated from data analysis are common day-to-day problems in microscopy research today, and the constantly growing size of image datasets does not help the matter. We propose a simple method and a software tool for sorting images within a dataset, according to their relative quality. We demonstrate the applicability of our method in finding good quality images in a STED microscope sample preparation optimization image dataset. The results are validated by comparisons to subjective opinion scores, as well as five state-of-the-art blind image quality assessment methods. We also show how our method can be applied to eliminate useless out-of-focus images in a High-Content-Screening experiment. We further evaluate the ability of our image quality ranking method to detect out-of-focus images, by extensive simulations, and by comparing its performance against previously published, well-established microscopy autofocus metrics.

Microscopists today have to deal with ever-growing image datasets. During a typical microscopy imaging session, tens or hundreds of images are regularly generated and (automatically) saved. Often only a handful of those images are useful for further analysis, and thus a lot of time is spent on searching for representative images. A single experiment on an automated High-Content-Screening (HCS) instrument may produce thousands of images. The results from such experiments need often to be converted straight into quantitative statistical measures; the quality of such measures strongly depends on the quality of the original data – *outliers* (e.g. out-of-focus images), may significantly compromise the results. There would thus, in our view, be a demand for a simple way to sort the images based on their qualitative content. Applications of such tool in imaging are almost endless: medical imaging, automated inspection, aerial and satellite imagery and bioimaging being just a couple of examples – not to forget the plethora of pictures and videos people capture and share online every day. It is therefore not a surprise, that computer-based objective image quality assessment is a rather popular research topic. Only a small number of publications however can be found on microscopy applications^1^,^2^,^3^, and to our knowledge, currently there are no applicable, easily accessible image quality assessment tools available for microscopy. Quantitative microscopy image analysis tools are being developed^4^,^5^,^6^,^7^, but for some reason, image quality assessment does not seem to be of great concern. This is rather surprising, because today’s microscopy techniques allow the realization of very ambitious experiments^8^,^9^,^10^, and sifting through terabytes of data manually is not really possible. The lack of such tools, in our view, can to a large part be explained by the fact that Bioimage informatics, the branch of Bioinformatics dealing with microscopy images, is still a rather nascent field – and many of the tools available in other disciplines e.g. Medical imaging, have not yet been realized for microscopy^11^,^12^,^13^.

Image quality assessment methods can be divided in several categories by their functionality. Often the quality of an image is estimated in relation to a reference image. These so-called *full reference* methods^14^,^15^ are used to estimate e.g. the performance of image compression methods, but they are of limited use in microscopy applications, as typically there is no reference image to be found. *Reduced reference*^16^,^17^ methods require knowledge of some characteristics of a good quality image. *No-Reference*^18^,^19^ methods try to estimate the image quality without a reference image or *a priori* standards – this would be the ideal setting for most practical applications, but it is also the most difficult from the point of the algorithm development. Many image quality estimation methods try to mimic the Human Visual System (HVS) by implementing complex mathematical models^18^,^19^. Machine-learning algorithms have also been implemented, in which before using the quality metric, the algorithm is first trained to recognize each distortion type in a training dataset that simulates the actual imaging situation^20^,^21^,^22^.

We propose an image quality ranking method, with no complicated mathematical model, no method training and no reference image. We show that practical image quality measures can be extracted from basic global statistics, in spatial and frequency domains. Furthermore, in our view, in many applications, rather than trying to estimate an absolute numerical value for image quality, it is sufficient to sort images within a given dataset according to their relative quality – hence the term image quality *ranking*. The purpose of such a ranking method is not to mimic human vision, but simply to aid in decision-making. While the definition of a good quality image is very application-specific and even subjective, there are some common characteristics that in our view, all good microscopy images should have, regardless of the application. A good fluorescent microscopy image should have contrast, bright details and dark background; ideally the whole dynamic range of the data acquisition device should be taken advantage of. A good quality image should also be properly focused.

In our ranking method, the image histogram is used to quantify image contrast, whereas details and blurriness are quantified with simple power-spectrum measures (such as mean, standard-deviation (STD) & skewness). The frequency domain measures are calculated from the high-frequency tail of the power-spectrum, in order to focus exclusively on finer image details, and to filter out the contribution of large spatial structures that can vary significantly from image-to-image; the frequency threshold value can be tuned to adjust the sensitivity of the spectral measures. Our concept of image quality ranking is based on simply taking one or e.g. average of several of the calculated statistical measures, and ordering the images based on its value. In order to make the different measures comparable, they were normalized, by dividing each measure by its maximum value within the processed dataset – all the measures thus got values between zero and one in any given dataset. With signed measures, such as skewness, absolute values were used. It should also be noted that blur should decrease the power spectral measures, whereas noise should do the opposite. Thus the measures should be inverted, when noise is of interest; in case the dataset consists both noisy and blurred images, the ranking should be run twice, once each way. This means that our method can be tuned to look for specific types of distortions (details) by choosing the appropriate measures and using them the right way.

From the results it can be seen that two image quality ranking measures can be combined to find images with good contrast and details, in a STED-microscopy dataset, derived from a sample preparation optimization experiment for labeling vimentin in BHK21 cells. The results are validated by comparisons to subjective opinion scores that were obtained by requesting microscopy experts to grade images, according to how good contrast and filamentous vimentin structure they have. In addition we compare the performance of our method against five state-of-the-art blind image quality assessment methods. We also show how our method can be applied to detect out-of-focus images within High-Content Screening (HCS) automated microscopy datasets. We further validate our results by comparing the blur detection ability of our image quality ranking method against previously published autofocus metrics, both with simulated data and with the real HCS image datasets.

## Results

### Investigating the power spectrum threshold

**Figure 1:**
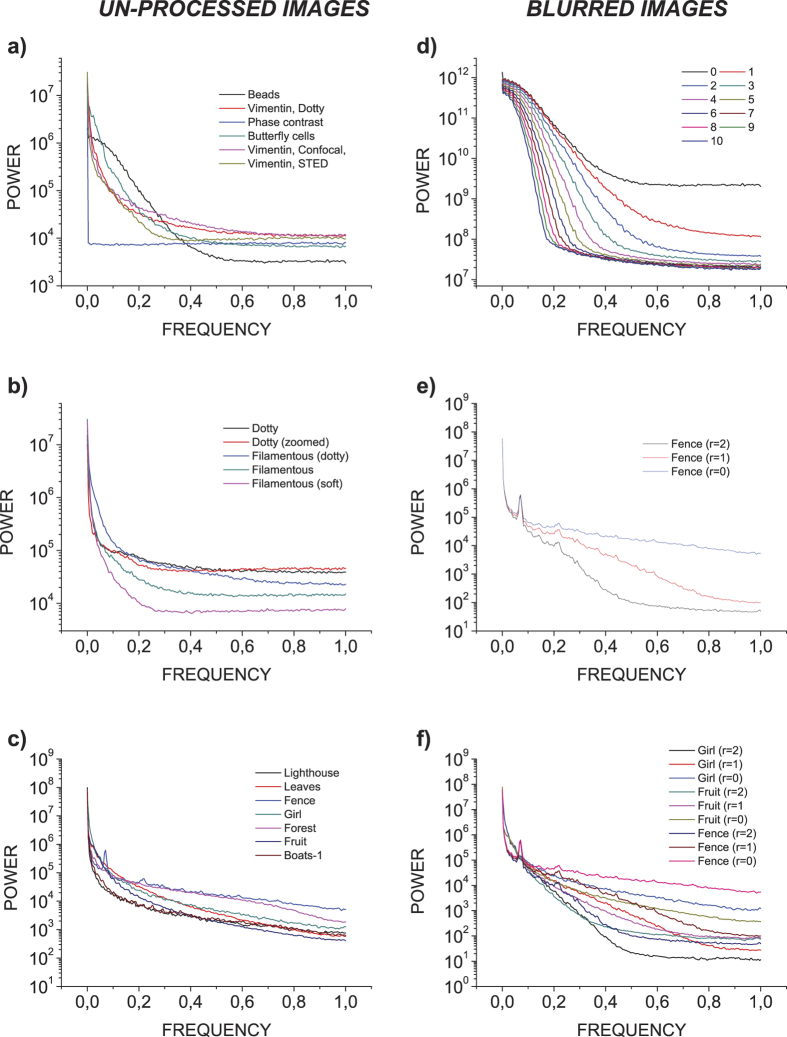
In (**a)** the power spectra of various kinds of microscopic images are shown. In (**b)** the power spectra of five images extracted from the STED sample preparation optimization dataset are shown. In (**c)** the power spectra of several regular photographs are shown. In (**d)** the power spectra are shown for a confocal image of fluorescent nanoparticles, Gaussian blurred with radii 0–10. In (**e)** the power spectra are shown of a single photograph, Gaussian blurred with radii 0–2. In (**f)** the similar spectra are shown of several photographs. All the photographs were selected form the simulation dataset, shown in (Supplementary Fig. 3). In all the sub-graphs Power denotes the normalized amount of signal power at a given frequency, whereas Frequency denotes the fraction of the maximum frequency in any given image.

Focusing on the high-frequency tail of the image power spectrum, should make it possible to calculate simple image quality related measures that are not sensitive to the large spatial structures, which change from one image to the next. In order to determine a suitable threshold value we investigated the power spectra of various kinds of un-processed as well as blurred microscope images and photographs. In (Fig. 1a) the power spectra of six very different microscope images are shown (Supplementary Fig. 4). As could be expected, the large spatial structures garble the different spectra at low frequencies, but at around 40% of the maximum frequency, it appears that the power spectra start to settle around a relatively fixed mean value and become quite clearly separable. Noisy images and images with abundant fine details seem to have a large amount of power at high frequencies, as could be expected. This observation is confirmed in (Fig. 1b) in which power spectra of five images (Supplementary Fig. 4) from the STED sample preparation optimization experiment are shown. Once again, there is significant variation at low frequencies, but after approximately 40% of the maximum frequency, the power spectra are clearly separate. Images with abundant fine details have a large amount of power at the high frequencies, whereas somewhat soft (blurred) images settle around a lower power value. Similar behavior can be observed in photographs (Fig. 1c), although the power spectra do not appear to settle around a mean value, but rather continue in a downward slope, up until the highest frequencies. Once again, at about 40% of the maximum frequency, the effect of the large spatial structures seems to disappear. In (Fig. 1d) the power spectra of Gaussian blurred versions (radii 0–10) of a single fluorescent nanoparticle image (Supplementary Fig. 4) are shown. Because each image is a version of a single base image, there is no mixing at low frequencies; it is also evident that in such case the images are easier to separate at the low frequencies, rather than high ones, and thus a low threshold value should be used. It also appears that it should not be necessary to calculate the whole power spectrum to separate the images, but rather a single bin near the zero frequency should suffice to separate the images. In blurred photographs (Fig. 1e,f) very similar behavior can be seen. Even a small amount of blur strongly affects the power spectrum tail. For this reason in-focus images, even from a mixture of different photographs (Fig. 1f) appear to be rather clearly separable from the blurred ones. Based on these observations, at approximately 40% of the maximum frequency of microscope images and photographs, it should be possible to calculate image quality ranking measures that are not affected by the variations of large spatial structures. Also, at the same threshold, the measures should be extremely sensitive to blur and noise. In autofocusing applications, it might be beneficial to decrease the threshold in order to increase the dynamic range of the blur detection.

### Detecting good quality images in a STED sample preparation optimization image dataset

**Figure 2:**
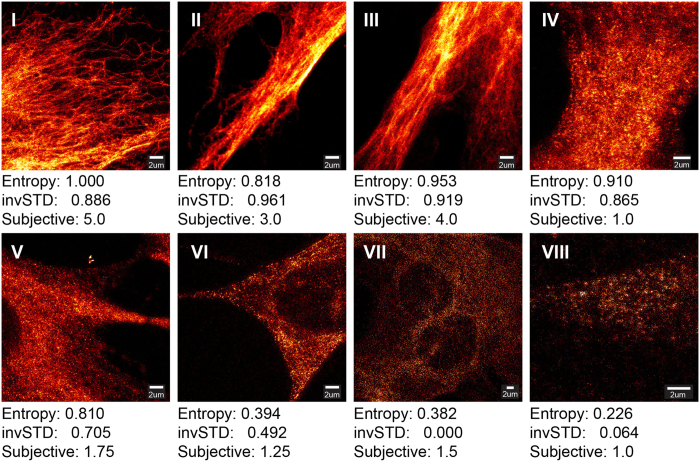
Four images both from the top and the bottom of the image quality ranking results of the STED microscopy dataset are shown . The masked spatial entropy (Entropy) measure correlates well with the image contrast, and the inverse of the frequency domain STD (invSTD) clearly favors images with good, non-dotty structures, as expected.

The ability of our method to recognize good quality images was tested by analyzing a STED microscopy dataset, containing images from a sample preparation optimization experiment for vimentin intermediate filaments in BHK21 cell-line (Supplementary Protocol 1). The aim was to devise a method for finding images with high contrast, low un-specific background signal and well visible, continuous spatial structure. To this end, the image ranking was performed, by averaging two measures: spatial entropy and inverse of the power spectrum STD (invSTD). The spatial entropy favors images with high contrast, whereas the invSTD measure should favor non-noisy images, as the dotty details that are typical to images from samples with less than optimal labeling, should amplify the power spectrum tail (Fig. 1b). In order to validate the ranking results, the performance of our method was compared against subjective opinion scores that were generated by microscope experts, who were asked to grade images with good contrast and filamentous vimentin structure on a scale 1–5. Moreover, the performance of our image quality ranking method was compared against five state-of-the art blind image quality assessment methods DIIVINE^23^, BRISQUE^24^, BLIINDS2^25^, NIQE^26^ & BIBLE^27^. As shown in (Fig. 2), both the Entropy and the invSTD measures correlate well with the observed image quality. However, as shown in (Fig. 2 image IV) the simple ranking measures cannot separate good filamentous vimentin structure, from dense and continuous structure not showing clear filaments. It is also evident that the subjective score of (Fig. 2 image IV), when seen side-by-side with images (Fig. 2 image V–VIII) appears rather severe. One can assume that the microscopy experts did not see filamentous structure, and thus gave a very low score to an otherwise good quality image; also, they were not allowed to view images side-by-side, but one at a time.

**Figure 3:**
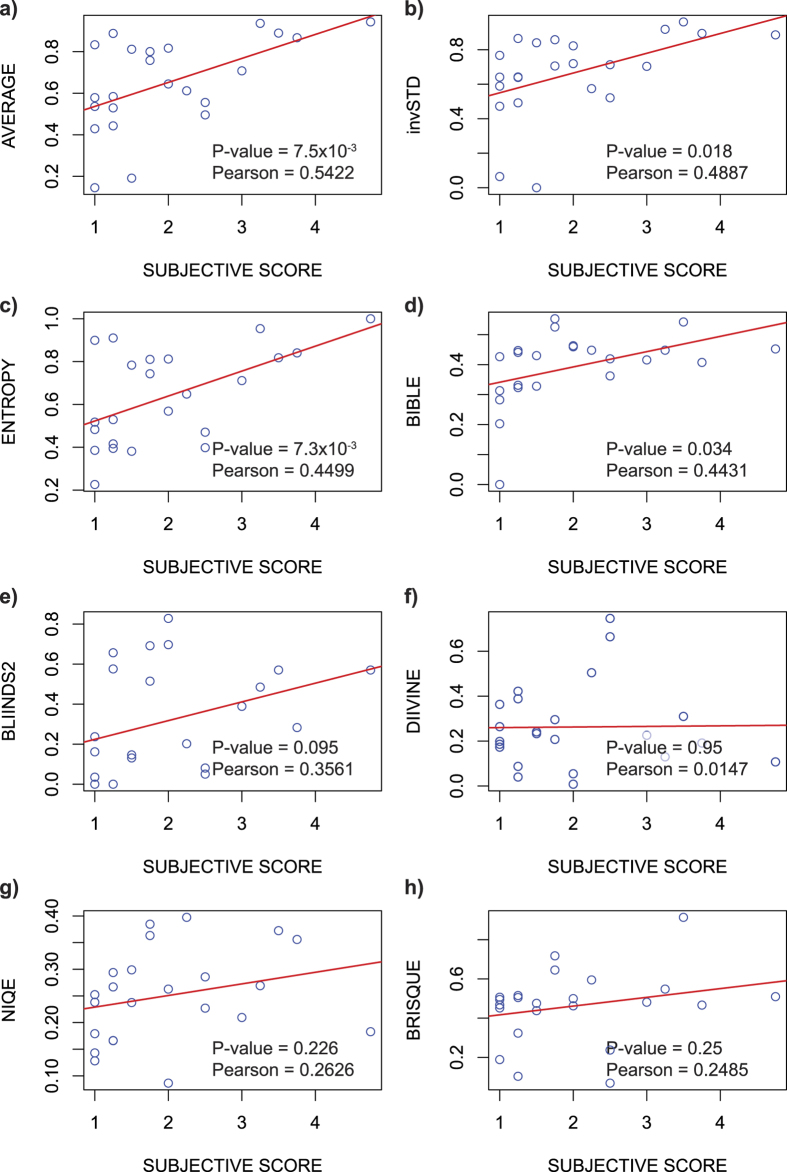
In (**a–c)** the correlation of various image quality ranking measures with subjective image quality scores are shown, when considering only STED images in the sample preparation optimization dataset. The term **Average** in (**a)** denotes the average of the invSTD & Entropy measures. In (**d–f)** corresponding plots are shown for each of the comparison image quality metrics. In the graphs the circles denote individual images and the red line is a linear regression fit of the data points. The quality of the linear model fit and Pearson correlation score is reported for each measure and metric.

In (Fig. 3) the correlation of the different image quality measures with the subjective scores is shown, in a subset of the STED image dataset that contains only STED images. The corresponding results for the complete dataset, containing a mixture of STED and confocal images, can be seen in (Supplementary Fig. 1). Both the invSTD and Entropy measures, as well as their combined Average correlate quite well with the subjective scores; the Average measure seems to be the best of the three. Subjective scores 2–5 match well with the ranking measures. However, there are several images that get a high ranking score, but a low subjective score – this can be attributed to the phenomenon shown in (Fig. 2): there are a lot of good quality images in the dataset that were given a very low subjective score, because they do not have evident filamentous vimentin structure. Separation of such images would require the implementation of structure-specific measures; one could e.g. look into the directionality of image gradients, which should be significantly different for dotty and filamentous images. Of the comparison methods, only BIBLE, BLIINDS2 and NIQE appear to correlate in any way with the subjective scores, BIBLE being clearly the best of the three. The same observations can be made with both the STED image subset and the complete dataset. In (Supplementary Fig. 2) the Average measure of our ranking method is compared against the five image quality assessment methods: BIBLE and BLIINDS2 correlate very nicely, NIQE to an extent, DIIVINE and BRISQUE not at all. It is probable that the natural image statistics that they estimate are not valid for microscopic images.

### Ranking blurred photographs

**Figure 4:**
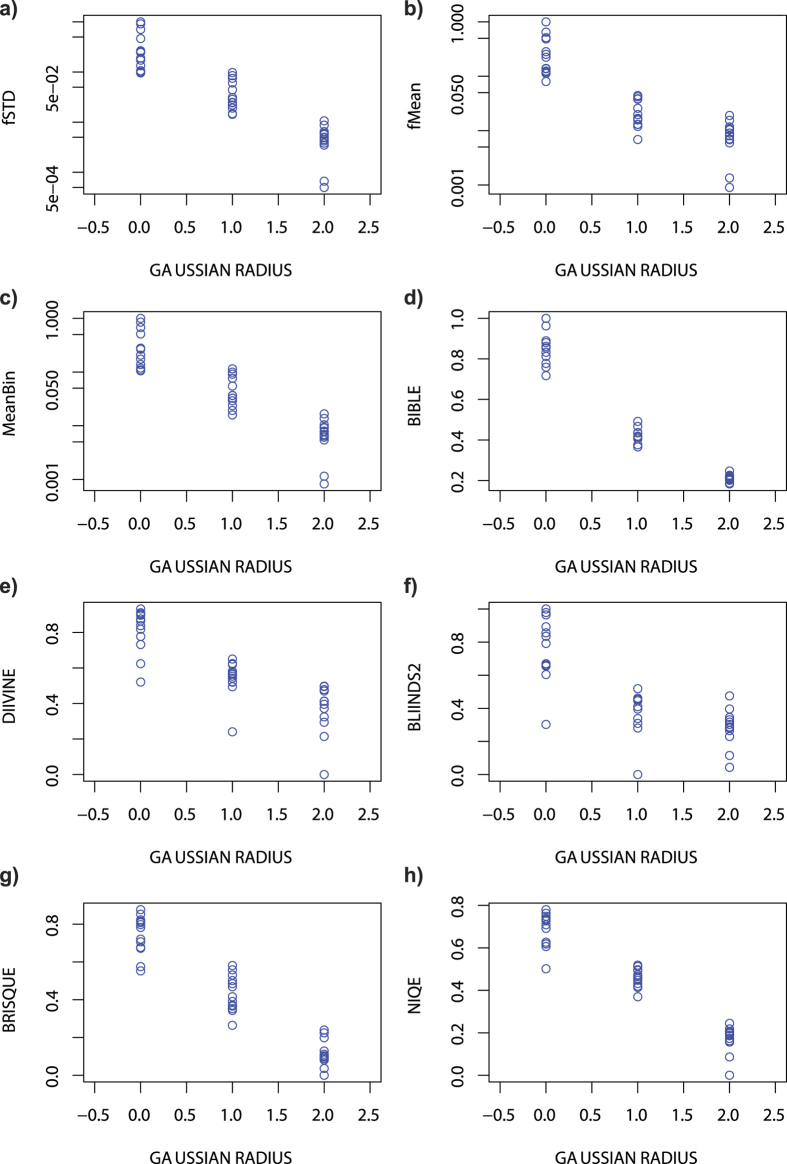
In (**a–c)** the values for fSTD, fMean and MeanBin are shown as a function of Gaussian blur radius in the photograph simulation dataset. In (**d–h)** Same kind of plots are shown for the benchmark image quality metrics BIBLE, DIIVINE, BLIINDS2, BRISQUE & NIQE. Notice that in (**a–c)** logarithmic scale is used, whereas linear scale is used in (**d–h)**.

A simulation dataset was created from a series of photographs (Supplementary Fig. 3), by applying Gaussian blur kernel of radii 0–2 to each of the images; the complete dataset is a mixture of all the images. Three frequency domain measures, spectral domain mean (fMean), standard-deviation (fSTD) and bin mean (MeanBin), of our ranking method were evaluated. MeanBin is the average power, calculated in a tiny five-sample-wide bin, starting from the selected threshold frequency, which was set to 40% from maximum, because it should make the measures very sensitive to blur, as suggested by the power spectra in (Fig. 1). As shown in (Fig. 4a–c), each measure responds rather aggressively to blur, making it possible to separate blurred images from originals, even in a mixed dataset such as this. fSTD and MeanBin produce the best separation, whereas fMean measure produces somewhat similar scores for Gaussian radii 1–2. It is quite impressive that the MeanBin measure manages to separate the mixed images well, by considering only a tiny subset of the power spectrum. We also compared the performance of our method against the five blind image quality assessment methods. Each of the five methods was developed to work on photographs, which should give them an advantage; surprisingly this is not so. DIIVINE and BLIINDS2 do not reliably separate original from even r = 2 blurred images. BRISQUE, NIQE and BIBLE all produce rather good results, BIBLE being clearly the best of the lot.

### Comparison of blur detection performance with autofocus metrics

**Figure 5:**
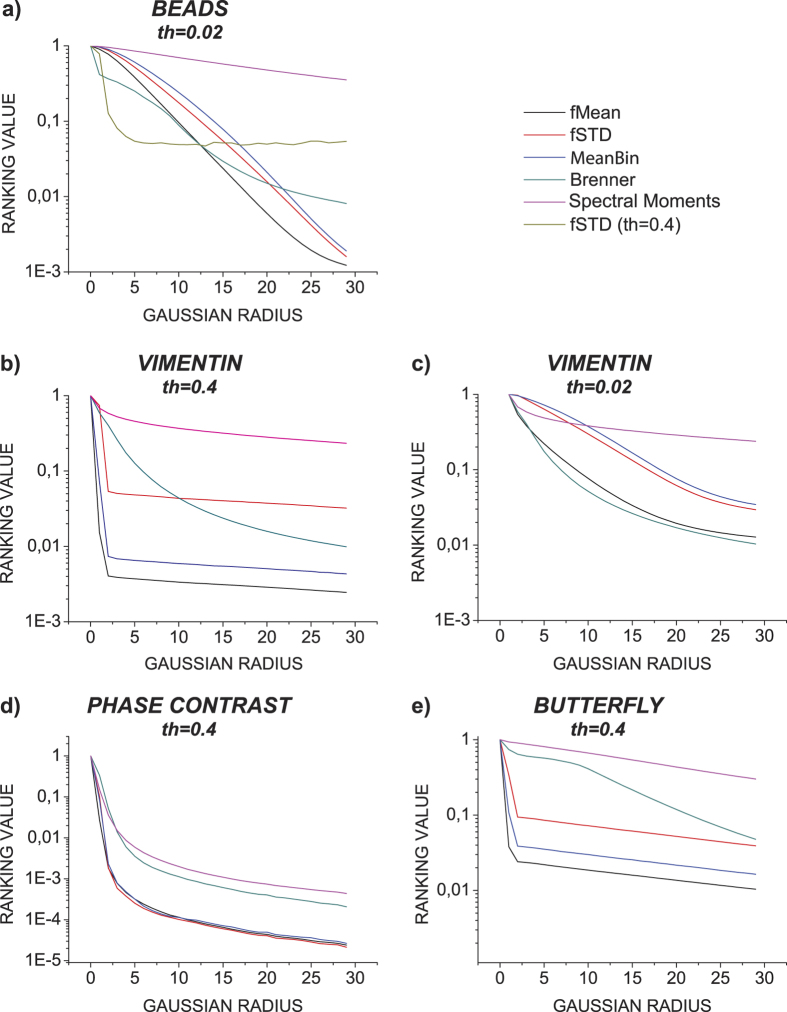
In (**a–e)** the performance of the fMean, fSTD & MeanBin measures is compared against Brenner and Spectral Moments autofocus metrics. The colors in each graph follow the legend shown on the upper right. The **th** denotes the value of the power spectrum threshold, that was used in calculating the fMean, fSTD & MeanBin measures. The base images that were used to create the blur series can be seen in (Supplementary Fig. 4).

Autofocus metrics in automated microscopy^28^ can be considered to be image quality ranking methods, but their use is typically limited to comparing differently blurred versions of the same image, whereas most microscopy datasets contain images from several positions on the sample object(s). We compared the blur detection performance of our image quality ranking method against two previously published, robust automatic microscopy autofocus metrics: the frequency domain based Spectral Moments metric^28^ and the spatial domain based Brenner metric^29^, which were both found excellent in simulations and with real images in^28^. The comparison was done on five different image datasets, each of which contain Gaussian blurred versions (radii 0–29) of four different microscope images: a phase contrast wide-field image (Phase Contrast), a high magnification confocal fluorescence image of intermediate filaments (Vimentin), a confocal fluorescence nanoparticle image (Beads) and a low-magnification image of rounded Butterfly cells (Butterfly). Several different images were used, in order to exclude the possibility that the measures react to some given spatial structure. The original images are shown in (Supplementary Fig. 4). Two different threshold values 40% and 2% were used in order to demonstrate the possibility to adjust the blur detection dynamic range in our ranking method. The same three ranking measures fSTD, fMean and MeanBin were used as with the photographs. In (Fig. 5a) the results from the nanoparticle focus series are shown. As could be expected from our power spectral observations (Fig. 1d), at 40% threshold the ranking works reliably only until Gaussian radius of approximately five. However, lowering the threshold to 2% completely linearizes the measures. At 2% threshold all the three measures clearly outperform the benchmark autofocus metrics; the Brenner metric works unreliably with the beads image, whereas Spectral Moments works well, but it does not have a very good sensitivity. The same effect is shown for the vimentin image series in (Fig. 5b,c). While in this case the ranking works at 40% as well, at 2% threshold the linearity is better. Once again, all the three ranking measures perform as well or better than the autofocus metrics. The three quality ranking measures work in a very similar manner with Phase contrast and Butterfly cell images as well (Fig. 5d,e), although phase-contrast images seem to be favored by all. Only the results for 40% threshold are shown here. The Spectral Moments autofocus metric seems to be rather reliable with all the test images, although it seems not to be very sensitive, except for with the Phase contrast image series. The Brenner metric appears unreliable with nanoparticle images, and surprisingly also with the Butterfly cell image series.

### Detecting out-of-focus images in HCS image datasets

**Figure 6:**
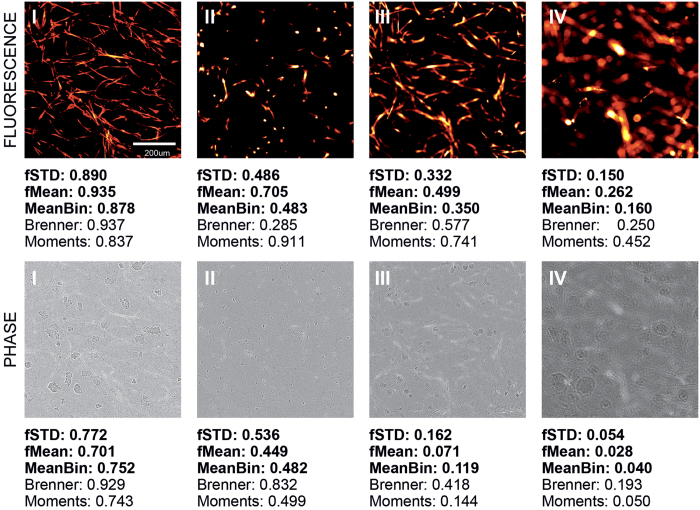
Examples of the image quality ranking results in the HCS image datasets are shown. The fSTD, fMean and MeanBin measures all clearly separate out-of-focus images from in-focus images with both fluorescence and phase-contrast. It is also possible to identify images, which are only slightly blurred. Especially good results were obtained with the phase-contrast images. Our quality ranking measures were also found more reliable than the two autofocus metrics. The four example images from the fluorescence and phase-contrast datasets represent (I) good, (II) nearly empty, (III) slightly blurred and (IV) clearly out-of-focus images.

We obtained two image datasets from an automated HCS time-course experiment of a 3D co-culture of LNCaP tumor cells together with PF179T stromal cells^30^ – each of the datasets contain a time series from a single well of a 96-well plate. The images were acquired in one image per well per hour fashion across the plate, and therefore auto-focusing was repeated for each image. In this type of an experiment the failure of autofocus function is one of the main concerns, as out-of-focus images corrupt the quantitative results. Our image quality ranking method was used to find the out-of-focus images from both of the datasets. The images contained phase-contrast and fluorescence channels, which were split into separate image series for the ranking. The three spectral domain measures fSTD, fMean and MeanBin that worked well in simulations were used here as well. The frequency threshold was set to 40%, because the image content in both fluorescent and phase channels varies significantly, and thus the low-frequency contribution needs to be filtered out. We also compared the performance of our ranking method against the two autofocus metrics, Brenner and Spectral Moments, which were used in the simulations earlier. Some examples from the ranking results are shown in (Fig. 6). Descriptive statistics for the measures were generated by manually browsing through the ranking results from one of the two datasets. The last clearly in-focus image was identified, after which the Mean and STD for each measure, above and below that point were calculated. The subjective review revealed that the fluorescence channel contained a large amount of images that are slightly blurred, although the corresponding phase-contrast images appear in focus. For this reason, for fluorescence images two thresholds were identified: one for the completely out-of-focus images, and another for these slightly blurred images. In both cases all the images below the threshold were identified as out-of-focus. The results are shown in (Table 1). In practical applications, filtering out the clearly out-of-focus images (Fig. 6 image IV) is the main interest, which means that the lower threshold should be used – raising the threshold too high might also cause the elimination of useful images, because in a time-course experiment, such as this one, the amount of image content varies significantly during the experiment, from nearly empty to detail packed, especially in the fluorescence channel (Fig. 6 images I–III). The nearly empty images typically get lower ranking values than the detail packed ones, despite our power spectral normalization.

With both of the datasets, using either fluorescence or phase-contrast, all out-of-focus images were found. All the three ranking measures worked well, surprisingly even the MeaBin measure. Both of the autofocus metrics worked rather well also. However, the Brenner method, being based on a form of spatial domain derivatives, suffered from the fact that not all the images in the dataset are versions of the one base image; images with less content, were mixed with the out-of-focus images in the fluorescence image datasets, whereas in the phase-contrast image datasets the out-of-focus images were mixed with only slightly blurred images and the nearly empty images received strangely high values. The Spectral Moments metric on the other hand worked quite well with the phase-contrast images, but it was quite insensitive to blur with the fluorescence images, although the clearly out-of-focus images were successfully identified – and for some reason the detail-less images received very high values. Our image quality ranking measures gave the most consistent results in both fluorescence and phase-contrast image datasets, and due to their strong response to small amounts of blur, they also successfully found only slightly blurred images, which were sometimes left unnoticed by the comparison methods. All the measures were more reliable with the phase-contrast images than the fluorescence ones. This result correlates well with the simulations.

## Discussion

A novel method for image quality ranking, aimed at microscopy applications was introduced. We demonstrated the applicability of our method to evaluating image quality within microscopy image datasets. We showed how the developed method could be used to find the good quality images in a STED microscopy dataset. The image quality ranking values correlated well with subjective scores. Our method also compared favorably against five state-of-the-art blind image quality assessment algorithms, also when using regular photographs, which is what those algorithms were created for. We also showed how the same method could be used to detect out-of-focus images in an automated HCS experiment. Similar applications can be found in all aspects of microscopy, and thus we think that the method would be of general interest to the bio-imaging/microscopy community. Applications could also be found in other fields of science, such as in medical imaging, automated inspection and aerial and satellite imagery.

Based on the simulations and results obtained with the HCS image datasets, our image quality ranking method could be quite powerful in an online autofocusing application as well. The possibility to tune the blur sensitivity by adjusting the power spectrum threshold would make it possible to create a highly accurate multi-stage focusing algorithm, in which the threshold is shifted up, as the image approaches perfect focus. Especially the MeanBin measure should be ideal in such an application, because it does not even require the calculation of the complete power spectrum and can be calculated very quickly. According to our results the performance of such a method should be at least at-par with current autofocus algorithms.

Although our image quality ranking method does not require complicated mathematical models, method training or reference image, additional features could of course be added, according to the requirements of a given application. One could for example use some form of method training to select a set of reference images, against which the other images would be compared. Also, integrating the quality ranking method with a pattern-recognition software^6^, would make it possible to select good quality images that in addition have the desired content, thus enabling a form of automatic image understanding^7^. Such a method could remove the offset between subjective opinion scores and ranking values that was seen in the STED image dataset.

Like any new image processing method, also ours has hope of finding applications only if there are available tools to try it with. To this end, we are releasing the source code of our own software *PyImageQualityRanking* under an open-source BSD license. The software was written in Python, using standard scientific libraries, available on all common operating systems, thus ensuring full cross-platform compatibility. There is also a plan to integrate our software with BioimageXD^31^ bio-image processing application in the future, in order to further improve the usability of our method^32^. Moreover, it is important to note that all of the functionality necessary to implement our method is available in practically all image processing software packages, as well as generic data analysis tools and programming languages. One could even contemplate implementing some of the functionality on an FPGA for autofocusing^28^, for example.

## Methods

### Contrast quantification by spatial domain histogram measurements

**Figure 7:**
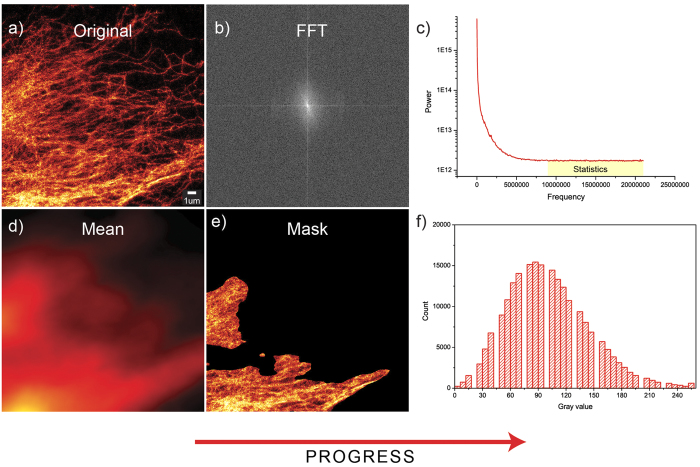
The working principle of the image-ranking tool is illustrated. In order to extract statistics related to image-structure, noise and blurriness, a frequency domain representation (**b**) of the original image (**a**) is computed via Fourier transform, after which it is simplified into a one-dimensional power-spectrum (**c**). All the frequency domain statistics are calculated only at the highest frequencies (typically >40% from maximum), as highlighted in (**c**). In order to compare the histograms of different images, a mask is first formed by filtering the original image (**a**) with a large (r = 100) uniform mean filter; the result of the operation can be seen in (**d**). By selecting the pixel positions in (**d**), at which the intensity is higher than 80% of the pixel values in the masked image, a spatial mask can be formed, to extract the neighborhoods in the original image that contain most details (**e**). Calculating the histogram from the masked image enables the comparison of images with varying amount of dark background.

The *histogram* is a powerful measure of image contrast in spatial domain. It represents the distribution of gray values within an image – a good quality image has ideally every single gray value within the dynamic range of the data acquisition device. We quantify the goodness of the histogram by Shannon entropy measure^20^,^33^,^34^:


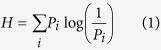


where *P*_*i*_denotes the normalized image histogram and *i* is the histogram bin index. Generally in fluorescence microscopy images, much of the field of view is black, and therefore the histograms tend to be dominated by the background values. To compare histograms of different images, the varying amount of background needs to be taken into account. We addressed this by calculating the histograms within masked image regions that had average intensity values significantly above the background level. The mask was created by first smoothing the image using a large (r = 100) averaging filter, and then thresholding it at 80^th^ percentile, which means that 80% of the pixels are below that value (see Fig. 7a,d–f). The averaging ensures that single bright spots (e.g. dust) do not get confused for image details. The 80% thresholding was determined experimentally by observing the content of the masked region in several test images (from the STED sample preparation optimization dataset): the aim was to select such a value at which only parts of the image containing useful details were included into the masked regions.

### Frequency domain analysis of image details

A spatial domain based histogram measure is limited to quantifying gray-scale contrast, whereas an image quality assessment method should take image sharpness/blurriness, noisiness and details into account as well. These properties can be quantified in the spatial domain by way of image segmentation or local filtering, but we decided to look into the power spectrum, derived from the frequency domain representation of an image, to find out simple descriptive statistics for image details. Our motivation for the spectral domain approach was that it should be more easily applicable to comparing similar images that are not different versions of a single base image. The spectral domain representations of images with similar structures can be expected to contain similar frequencies, although their spatial organization might differ significantly. A power spectrum measurement should thus to some extent be applicable to evaluating image content, and more importantly it can be used to detect blur and noise: blurriness should decrease the amount of fine details and thus attenuate high frequencies, whereas noisiness should do the opposite^18^,^35^. We therefore decided to focus exclusively on the fine details (high frequencies), by calculating a number of statistical measures, such as mean, standard deviation (STD) and skewness of the power spectrum tail (Fig. 7a–c). Inverted measures can also be used, when noise is of interest; in case the dataset consists both noisy and blurred images, the ranking should be run twice, once each way. This means that our method can be tuned to look for specific types of distortions (details) by choosing the appropriate measures and using them the right way.

The power spectral analysis of an image was performed, as follows. First, a two-dimensional power spectrum image was calculated by taking a square of the centered frequency-domain image, thus precluding negative power values. The effect of image-to-image brightness (content) variation was addressed by dividing the power spectrum by the image average gray level and the total number of pixels, similar to what was done in^18^. A one-dimensional power-spectrum was then calculated, by taking advantage of one of the two implemented methods: 1° by calculating an average power amplitude at a given radius from the zero frequency center or 2° by first adding all the rows and columns together and then adding the corresponding “negative” frequencies to the “positive” ones. The second method is significantly faster to calculate, as it only consists of additions and was thus used throughout our experiments. In order to focus exclusively on fine image details, the power spectrum was then cropped to contain only frequencies larger than a set threshold value, and then the following statistics were calculated on the power spectrum tail: mean (fMean), standard-deviation (fSTD), cv, kurtosis, entropy, skewness and summed power amplitude at frequencies larger than 90% of the maximum frequency. MeanBin parameter is the average power, calculated at a single five-sample-wide bin, starting from the selected threshold. Also inverse measures were calculated for skewness and STD, i.e. the invSTD measure value equals to 1-STD; similar measures could be calculated from e.g. MeanBin and fSTD.

### STED microscopy dataset

In order to optimize a sample preparation protocol for vimentin intermediate filaments in BHK21 cells, samples were prepared with a combination of different fixation, permeabilization and blocking methods. Two different primary antibodies, V9 (Sigma) and D21H3 (Cell Signaling Technologies) were applied to each sample preparation method. Two different secondary antibodies, Atto647N (Invitrogen) and Abberior Star635P (Abberior), were used as well (Supplementary Protocol 1). The samples were imaged with a Leica TCS STED (Leica Microsystems) super-resolution microscope. The STED microscopy image dataset contains a mixture of STED and confocal images, at various zoom levels; the STED images can be separated from the confocal images using the file filtering functionality in our *PyImageQualityRanking* software (see *Image Quality Ranking Software*).

### Simulation photograph dataset

Twelve grayscale photographs were used to create a simulation dataset (Supplementary Fig. 3). Two blurred versions of each of the pictures were created, by using a Gaussian blur filter, with radii 1.0 and 2.0. The image dataset was generated with a custom Python script that is included in our PyImageQualityRanking software (see *Image Quality Ranking Software*).

### Autofocus simulation datasets

Four microscope auto-focus simulation datasets were generated from Vimentin, Beads, Phase Contrast & Butterfly Cells images (Supplementary Fig. 4). Each base image was Gaussian blurred with radii 0–29 in order to create simulated focus series. The image datasets were generated with a custom Python script that is included in our PyImageQualityRanking software (see. *Image Quality Ranking Software*).

### HCS time series datasets

Two datasets from an automated HCS time-course experiment of a 3D co-culture of LNCaP tumor cells together with PF179T stromal cells (see^30^ for full details) were used to test the possibility to detect out-of-focus images with our image quality ranking method. Each of the datasets contains a time series from a single well of a 96-well plate, a little over 300 images, with both phase-contrast and fluorescence channels. In total there were thus approximately 1200 images to be ranked.

### Subjective image quality assessment

Subjective scores for the STED microscope image dataset (see *STED microscopy dataset*) were obtained by asking microscopy experts to rank the images on a scale “Bad”, “Poor”, “Fair”, “Good”, “Excellent” (numeric 1–5)^15^,^36^. The experts were requested to identify images with good contrast and nice, fibrous intermediate filament structure. The images were evaluated in one-by-one fashion, not side-by side. At each re-run the order of the images was shuffled. The opinion scores used in in the results section are the average of four subjective rankings. The subjective image ranking was performed with the help of a custom Python script that is included in our PyImageQualityRanking software (see *Image Quality Ranking Software*).

### Method validation against blind image quality assessment methods

Our image quality ranking method was compared against five state-of-the-art blind image quality assessment methods: DIIVINE^23^, BRISQUE^24^, BLIINDS2^25^, NIQE^26^ & BIBLE^27^. Our requirements for the comparison methods were that they should not require a reference image, and that a functioning reference software implementation that can be used for testing, has been made available by the authors. DIIVINE, BRISQUE and BLIINDS2 are so called opinion-aware blind image quality assessment methods, which means that they have been trained to recognize different kinds of distortions by using images with known distortion type and human opinion score. NIQE and BIBLE are completely blind methods, i.e. they do not require method training. BIBLE is specifically aimed at detecting image blur, whereas the other four methods are aimed at general image quality assessment. Our tests were done on the original software released by the authors; for each of the five methods Matlab code was either directly available online, or upon request. No changes were made to the original implementations. DIIVINE, BRISQUE and BLIINDS2 were supplied pre-trained to recognize all common image degradations. The comparisons were done with the STED microscope image dataset (see *STED microscopy dataset*) and the blurred photograph simulation dataset (see. *Simulation photograph dataset*). Same kind of normalization was used as with our own ranking measures, in order to establish a common (0–1) scale for the image quality measures.

### Method validation against autofocus metrics

We compared the blur detection performance of our image quality ranking method against two previously published, robust automatic microscopy autofocus metrics. The Brenner metric^29^ is a form of a spatial domain derivative:


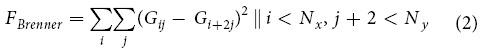


where G_ij_ is the grayscale intensity at pixel position ij, N_*x*_ and N_y_ are the image width and height. It has been shown to be a very robust autofocus metric^28^. The Spectral moments metric^28^ is calculated from the image power spectrum, similarly to what is done in our image quality ranking method:


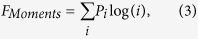


where i is the spectral component index and P is the normalized power spectrum 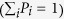. However, the entire power spectrum is used for the metric calculation, instead of cropping it to contain only the high frequencies, as is done in our method – the logarithmic scaling does somewhat emphasize high frequencies. We implemented the two autofocus metrics in our *PyImageQualityRanking* software (see *Image quality ranking software*).

### Image Quality Ranking Software

Our *PyImageQualityRanking* software was written in Python, and it is released under BSD open-source license. Only standard Scientific Python libraries (Numpy, SciPy, Pandas, Matplotlib) are utilized. Numpy and SciPy were used to implement all the image processing and analysis features, whereas Pandas was used to implement methods to process the results (data sorting by measure value, measure calculations, measure normalization etc.). Matplotlib was used for image visualization, as well as to produce mathematical plots. In many quantitative microscopy applications time lapse recordings are made, which means that the order of the images should not be changed – therefore the data sorting features in *PyImageQualityRanking* software do not actually change the order of the images, but instead the software creates a separate data file with the image names and measures, which can be safely modified, without risk of compromising the original image data. The basic functionality of the *PyImageQualitRanking* software is described in pseudocode in (Supplementary Note 1) .

## Additional Information

**How to cite this article**: Koho, S. *et al*. Image Quality Ranking Method for Microscopy. *Sci. Rep.* **6**, 28962; doi: 10.1038/srep28962 (2016).

## Supplementary Material

Supplementary Information

## Figures and Tables

**Table 1:** Statistics from the HCS dataset ranking are shown for each measure.

	fSTD	fMean	MeanBin	Brenner	Spectral Moments
Phase Contrast					
In-Focus	0.76/0.1	0.72/0.1	0.73/0.1	0.93/0.03	0.74/0.1
Out-of-focus	0.12/0.08	0.05/0.03	0.09/0.06	0.32/0.15	0.10/0.06
Fluorescence					
In-Focus	0.68/0.11	0.8/0.1	0.69/0.10	0.81/0.15	0.83/0.04
Out-of-focus	0.20/0.04	0.34/0.04	0.21/0.04	0.21/0.05	0.45/0.08
Blurred	0.38/0.1	0.56/0.13	0.39/0.10	0.37/0.16	0.77/0.17

In the Fluorescence channel two levels of blurriness were identified: clearly out-of-focus images, and in-focus, but clearly blurred images. Therefore two levels of blurriness are reported here. The numbers denote (Mean/STD) of all the values above and below the last in-focus image.

## References

[b1] Bray M.-A., Fraser A. N., Hasaka T. P. & Carpenter A. E. Workflow and Metrics for Image Quality Control in Large-Scale High-Content Screens. J. Biomol. Screen. 17, 266–274 (2012). 21956170 10.1177/1087057111420292 PMC3593271

[b2] Redondo R. . Quality evaluation of microscopy and scanned histological images for diagnostic purposes. Micron 43, 334–343 (2012). 22099388 10.1016/j.micron.2011.09.010

[b3] Paul P., Kalamatianos D., Duessmann H. & Huber H. Automatic quality assessment for fluorescence microscopy images. In 8th IEEE International Conference on BioInformatics and BioEngineering, doi: 10.1109/BIBE.2008.4696665 (IEEE, 2008).

[b4] Ljosa V. & Carpenter A. E. Introduction to the Quantitative Analysis of Two-Dimensional Fluorescence Microscopy Images for Cell-Based Screening. PLos Comput. Biol. 5, e1000603 (2009). 20041172 10.1371/journal.pcbi.1000603 PMC2791844

[b5] Shariff A., Kangas J., Coelho L. P., Quinn S. & Murphy R. F. Automated Image Analysis for High-Content Screening and Analysis. J. Biomol. Screen. 15, 726–734 (2010). 20488979 10.1177/1087057110370894

[b6] Shamir L., Delaney J. D., Orlov N., Eckley D. M. & Goldberg I. G. Pattern Recognition Software and Techniques for Biological Image Analysis. PLos Comput Biol 6, e1000974 (2010). 21124870 10.1371/journal.pcbi.1000974 PMC2991255

[b7] Huang K. & Murphy R. F. From Quantitative Microscopy to Automated Image Understanding. J. Biomed. Opt. 9, 893–912 (2004). 15447010 10.1117/1.1779233 PMC1458526

[b8] Keller P. J., Schmidt A. D., Wittbrodt J. & Stelzer E. H. K. Digital Scanned Laser Light-Sheet Fluorescence Microscopy (DSLM) of Zebrafish and Drosophila Embryonic Development. Cold Spring Harb. Protoc. 2011, pdb.prot065839 (2011). 21969622 10.1101/pdb.prot065839

[b9] Wang K. . Rapid adaptive optical recovery of optimal resolution over large volumes. Nat. Methods 11, 625–628 (2014). 24727653 10.1038/nmeth.2925 PMC4069208

[b10] Chen B.-C. . Lattice light-sheet microscopy: Imaging molecules to embryos at high spatiotemporal resolution. Science 346, 1257998 (2014). 25342811 10.1126/science.1257998 PMC4336192

[b11] Myers G. Why bioimage informatics matters. Nat. Methods 9, 659–660 (2012). 22743769 10.1038/nmeth.2024

[b12] The quest for quantitative microscopy (Editorial). Nat. Methods 9, 627–627 (2012). 22930824 10.1038/nmeth.2102

[b13] Eliceiri K. W. . Biological imaging software tools. Nat. Methods 9, 697–710 (2012). 22743775 10.1038/nmeth.2084 PMC3659807

[b14] Wang Z., Bovik A. C., Sheikh H. R. & Simoncelli E. P. Image quality assessment: from error visibility to structural similarity. IEEE Trans. Image Process. 13, 600–612 (2004). 15376593 10.1109/tip.2003.819861

[b15] Sheikh H. R. & Bovik A. C. Image information and visual quality. IEEE Trans. Image Process. 15, 430–444 (2006). 16479813 10.1109/tip.2005.859378

[b16] Wang Z. & Bovik A. C. Reduced- and No-Reference Image Quality Assessment. IEEE Signal Process. Mag. 28, 29–40 (2011).

[b17] Soundararajan R. & Bovik A. C. RRED Indices: Reduced Reference Entropic Differencing for Image Quality Assessment. IEEE Trans. Image Process. 21, 517–526 (2012). 21878414 10.1109/TIP.2011.2166082

[b18] Nill N. B. & Bouzas B. Objective image quality measure derived from digital image power spectra. Opt. Eng. 31, 813–825 (1992).

[b19] Ferzli R. & Karam L. J. A No-Reference Objective Image Sharpness Metric Based on the Notion of Just Noticeable Blur (JNB). IEEE Trans. Image Process. 18, 717–728 (2009). 19278916 10.1109/TIP.2008.2011760

[b20] Liu L., Liu B., Huang H. & Bovik A. C. No-reference image quality assessment based on spatial and spectral entropies. Signal Process. Image Commun. 29, 856–863 (2014).

[b21] Narwaria M. & Lin W. Objective Image Quality Assessment Based on Support Vector Regression. IEEE Trans. Neural Netw. 21, 515–519 (2010). 20100674 10.1109/TNN.2010.2040192

[b22] Narwaria M. & Lin W. SVD-Based Quality Metric for Image and Video Using Machine Learning. IEEE Trans. Syst. Man Cybern. Part B Cybern. 42, 347–364 (2012). 10.1109/TSMCB.2011.2163391 21965214

[b23] Moorthy A. K. & Bovik A. C. Blind Image Quality Assessment: From Natural Scene Statistics to Perceptual Quality. IEEE Trans. Image Process. 20, 3350–3364 (2011). 21521667 10.1109/TIP.2011.2147325

[b24] Mittal A., Moorthy A. K. & Bovik A. C. No-Reference Image Quality Assessment in the Spatial Domain. IEEE Trans. Image Process. 21, 4695–4708 (2012). 22910118 10.1109/TIP.2012.2214050

[b25] Saad M. A., Bovik A. C. & Charrier C. Blind Image Quality Assessment: A Natural Scene Statistics Approach in the DCT Domain. IEEE Trans. Image Process. 21, 3339–3352 (2012). 22453635 10.1109/TIP.2012.2191563

[b26] Mittal A., Soundararajan R. & Bovik A. C. Making a ‘Completely Blind’ Image Quality Analyzer. IEEE Signal Process. Lett. 20, 209–212 (2013).

[b27] Li L. . No-Reference Image Blur Assessment Based on Discrete Orthogonal Moments. IEEE Trans. Cybern. 46, 39–50 (2016). 25647763 10.1109/TCYB.2015.2392129

[b28] Firestone L., Cook K., Culp K., Talsania N. & Preston K. Comparison of autofocus methods for automated microscopy. Cytometry 12, 195–206 (1991). 2036914 10.1002/cyto.990120302

[b29] Brenner J. F. . An automated microscope for cytologic research a preliminary evaluation. J. Histochem. Cytochem. Off. J. Histochem. Soc. 24, 100–111 (1976). 10.1177/24.1.1254907 1254907

[b30] Åkerfelt M. . Automated tracking of tumor-stroma morphology in microtissues identifies functional targets within the tumor microenvironment for therapeutic intervention. Oncotarget 6, 30035–30056 (2015). 26375443 10.18632/oncotarget.5046 PMC4745780

[b31] Kankaanpää P. . BioImageXD: an open, general-purpose and high-throughput image-processing platform. Nat. Methods 9, 683–689 (2012). 22743773 10.1038/nmeth.2047

[b32] Carpenter A. E., Kamentsky L. & Eliceiri K. W. A call for bioimaging software usability. Nat. Methods 9, 666–670 (2012). 22743771 10.1038/nmeth.2073 PMC3641581

[b33] Tsai D.-Y., Lee Y. & Matsuyama E. Information Entropy Measure for Evaluation of Image Quality. J. Digit. Imaging 21, 338–347 (2008). 17577596 10.1007/s10278-007-9044-5 PMC3043833

[b34] Gabarda S. & Cristóbal G. Quality evaluation of blurred and noisy images through local entropy histograms. In Proc. SPIE 6592, Bioengineered and Bioinspired Systems III, 659214, doi: 10.1117/12.721952 (SPIE, 2007).

[b35] Field D. J. & Brady N. Visual sensitivity, blur and the sources of variability in the amplitude spectra of natural scenes. Vision Res. 37, 3367–3383 (1997). 9425550 10.1016/s0042-6989(97)00181-8

[b36] Sheikh H. R., Sabir M. F. & Bovik A. C. A Statistical Evaluation of Recent Full Reference Image Quality Assessment Algorithms. IEEE Trans. Image Process. 15, 3440–3451 (2006). 17076403 10.1109/tip.2006.881959

