# Erratum: Image Quality Ranking Method for Microscopy

**DOI:** 10.1038/srep30863

**Published:** 2016-08-19

**Authors:** Sami Koho, Elnaz Fazeli, John E. Eriksson, Pekka E. Hänninen

Scientific Reports 6: Article number: 2896210.1038/srep28962; published online: 07 01 2016; updated: 08 19 2016.

In this Article, the *PyImageQualityRanking* software source code has been omitted from the Methods section under subheading ‘Image Quality Ranking Software’. It should read:

“The *PyImageQualityRanking* software’s source code can be downloaded from https://bitbucket.org/sakoho81/pyimagequalityranking/. The software is distributed as a standard Python package, which contains an automatic setup script for easy installation. A convenient command line interface is provided for controlling various measures. For further details, please refer to the software documentation at https://bitbucket.org/sakoho81/pyimagequalityranking/wiki/Home.”

