# Supplementary material for: Image Quality Ranking Method for Microscopy
Source: Sci Rep. 2016 Jul 1;6:28962. doi: 10.1038/srep28962 (PMC4929473; doi:10.1038/srep28962)
Supplement: Supplementary Information [file srep28962-s1.pdf]

Supplementary material for:

Image Quality Ranking Method for Microscopy

Sami Koho, Elnaz Fazeli, John E. Eriksson & Pekka E. Hänninen

**Reference**	**Description**
Supplementary Protocol 1	The sample preparation protocol for labeling vimentin in BHK21 cells for STED super-resolution microscopy is explained
Supplementary Figure 1	The correlation of various image quality ranking measures with subjective image quality scores are shown, when simultaneously considering the STED and confocal images in the sample preparation optimization dataset.
Supplementary Figure 2	The results of our image quality ranking method are compared with the BIBLE, BLIINDS2, BRISQUE, DIIVINE & NIQE benchmark image quality metrics in the complete sample preparation optimization STED/confocal dataset.
Supplementary Figure 3	The original images that were used to create the simulation photograph dataset are shown
Supplementary Figure 4	The microscopic images that were used to generate the power spectrum plots, as well as the autofocus simulation datasets are shown.
Supplementary Note 1	The basic functionality of the *PyImageQualityRanking* software is described in pseudocode.

Supplementary Protocol 1: STED sample preparation protocol for Vimentin in BHK21 cells

Aims

- Compare different vimentin primary antibodies to see which produces the best STED images
- Compare different secondary antibodies for STED imaging
- Find the best fixation, permeabilization, blocking method, primary and secondary antibody combinations for STED imaging to obtain the best quality images

Cell culture

BHK21 cells were cultured in 100x20 mm cell culture dishes (BD Falcon) using Dulbecco's Modified Eagle Medium (SMEM; GIBCO/Sigma), and were incubated in in 5% CO_2 humidified atmosphere. The cells were divided after two days into twelve well plates (CellStar), placing 15000 cells in every well on top of microscope coverslips (ϕ16mm, #1.5 Menzel gläser).

Fixation, permeabilization and blocking

Samples were prepared in a number of ways, using different combinations of fixatives, permeabilizers and blockers:

1 **PFA (Paraformaldehyde), TritonX, BSA (Bovine Serum Albumin)**
Coverslips were washed with 1x concentration of PBS (Phosphate Buffered Saline). The cells were fixed using 3.7% PFA and incubated for 5 minutes at room temperature. Afterwards the coverslips were washed with 1x PBS and cells were permeabilized with 0.1% Triton-X solution in PBS for 5 minutes at room temperature. Washing step was repeated. Subsequently cells were blocked with 1% BSA solution in PBS and then incubated for one hour at room temperature.

2 **PFA, TritonX, Goat serum**
The protocol is the same as described above. However 10% goat serum (Invitrogen) in PBS was used in the blocking step instead of BSA.

3 **PFA, TritonX, Fish skin gelatin**
The protocol is the same as described above. However fish skin gelatin solution was used in the blocking step instead of BSA

Fish skin gelatin solution (Sigma) is composed of 222 µl of 45% fish skin gelatin, 0.1 g non-fat powdered milk and 0.1 ml goat serum in 10 ml PBS

4 Methanol, BSA

Coverslips were washed with 1x concentration of PBS. Then the cells were fixed using 100% Methanol and incubate for 7 minutes at -20 °C. Consequently cells were blocked with 1% BSA solution in PBS and incubated for one hour at room temperature.

5 Methanol, Goat serum

The protocol is the same as described above. However 10% goat serum-PBS was used in the blocking step instead of BSA.

6 Methanol, Fish skin gelatin

The protocol is the same as described above. However fish skin gelatin solution was used in the blocking step instead of BSA

7 Methanol, Acetone, BSA

Coverslips were washed with 1x concentration of PBS. The cells were fixed using 100% Methanol and incubated for 7 minutes at -20 °C. The coverslips were washed with 1x PBS, after which the cells were permeabilized with 100% Acetone for 1 minute at -20 °C. Washing step was repeated. The cells were blocked with 1% BSA solution in PBS and incubated for one hour at room temperature.

8 Methanol, Acetone, Goat serum

The protocol is the same as described above. However 10% goat serum-PBS was used in the blocking step instead of BSA

9 Methanol, Acetone, Fish skin gelatin

The protocol is the same as described above. However fish skin gelatin solution was used in the blocking step instead of BSA

10 Acetone, BSA

Coverslips were washed with 1x concentration of PBS. Then the cells were fixed using 100% Acetone and incubated for 7 minutes at -20 °C. Afterwards the coverslips were washed with 1x PBS. Consequently the cells were blocked with 1% BSA solution in PBS and incubated for one hour at room temperature.

11 Acetone, Goat serum

The protocol is the same as described above. However 10% goat serum-PBS was used in the blocking step instead of BSA

12 Acetone, Fish skin gelatin

The protocol is the same as described above. However fish skin gelatin solution was used in the blocking step instead of BSA

13 Methanol: Acetone, BSA

Coverslips were washed with 1x concentration of PBS. Then the cells were fixed using 1:1 solution that consisted of 100% Methanol and 100% Acetone, and then incubated for 7 minutes at -20 °C. Afterwards the coverslips were washed with 1x PBS. Consequently the cells were blocked with 1% BSA solution in PBS, and incubated for one hour at room temperature.

14 Methanol: Acetone, Goat serum

The protocol is the same as described above. However 10% goat serum-PBS was used in the blocking step instead of BSA

15 Methanol: Acetone, Fish skin gelatin

The protocol is the same as described above. However fish skin gelatin solution was used in the blocking step instead of BSA

Labeling

Two different primary (V9 and D21H3) and secondary (Atto647N & Abberior Star 635P) antibodies were used for each fixation, permeabilization and blocking combination. In addition, different concentrations of secondary antibodies were compared (1:500, 1:750 and 1:1000).

The labeling was done as follows:

1. Addition of the primary antibody (1:100 concentration)
2. Incubation at +4 ºC overnight,
3. Washing by dipping three times in 1x PBS.
4. Addition of the secondary antibody
5. Incubation at room temperature for 45 min at room temperature.
6. Washing by dipping coverslips twice into 1x PBS and once in MQ-H_2O.
7. Mounting with Mowiol

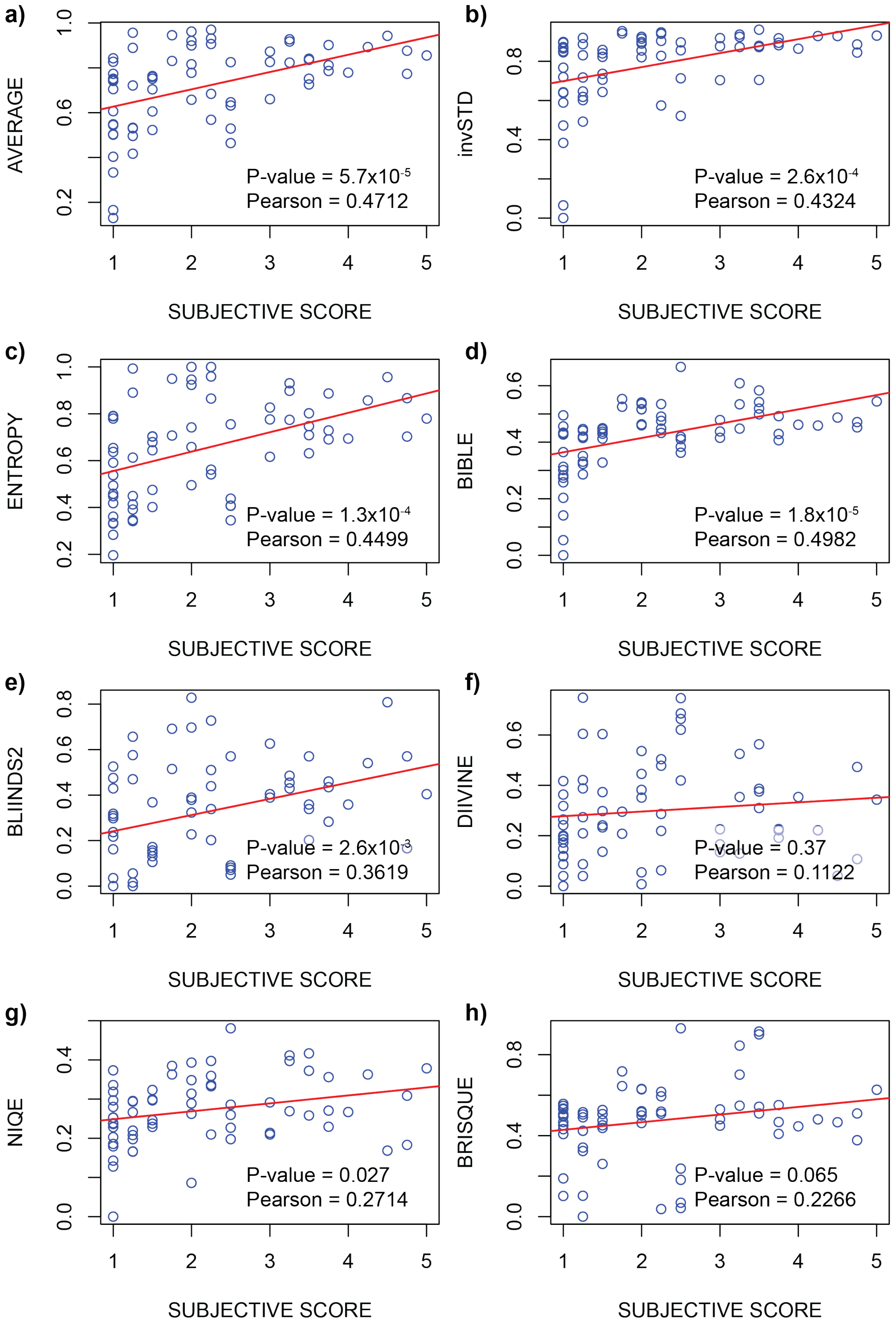

*Supplementary Figure 1: In **a)** the correlation of various image quality ranking parameter values with subjective image quality scores are shown, when simultaneously considering the STED and confocal images in the sample preparation optimization dataset. The term **Average** in **a)** denotes the average of the invSTD & Entropy parameters. In **d-f)** corresponding plots are shown for each of the comparison image quality metrics. In the graphs the circles denote individual images and the red line is a linear regression fit of the data points. The quality of the linear model fit and Pearson correlation score is reported for each parameter and metric.*

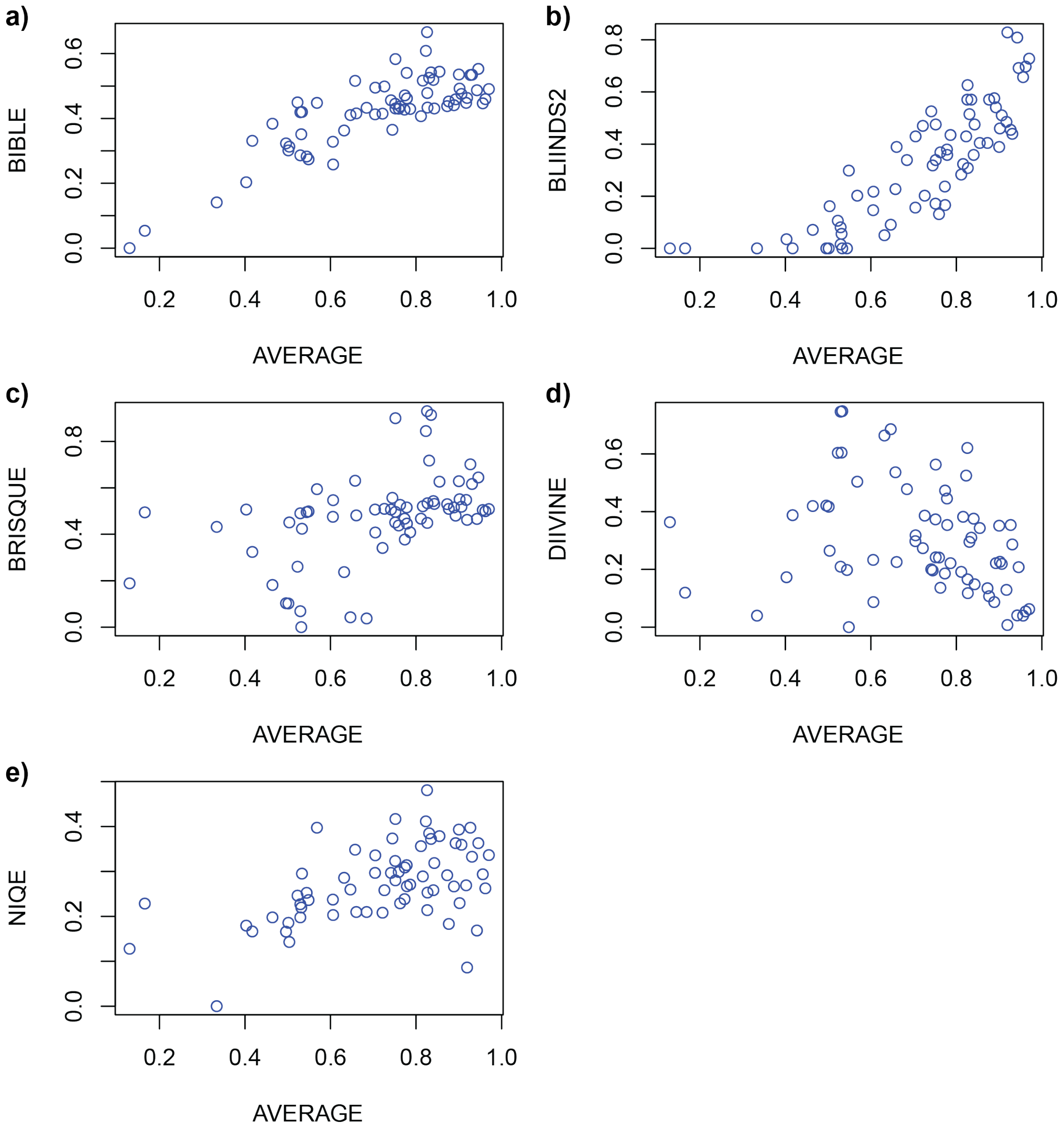

*Supplementary Figure 2: In **a-e)** the values of the AVERAGE parameter (average of invSTD and spatial Entropy) of our image quality ranking method, are compared with the BIBLE, BLIINDS2, BRISQUE, DIIVINE & NIQE benchmark image quality metrics. Each circle denotes an image in the sample preparation optimization dataset.*

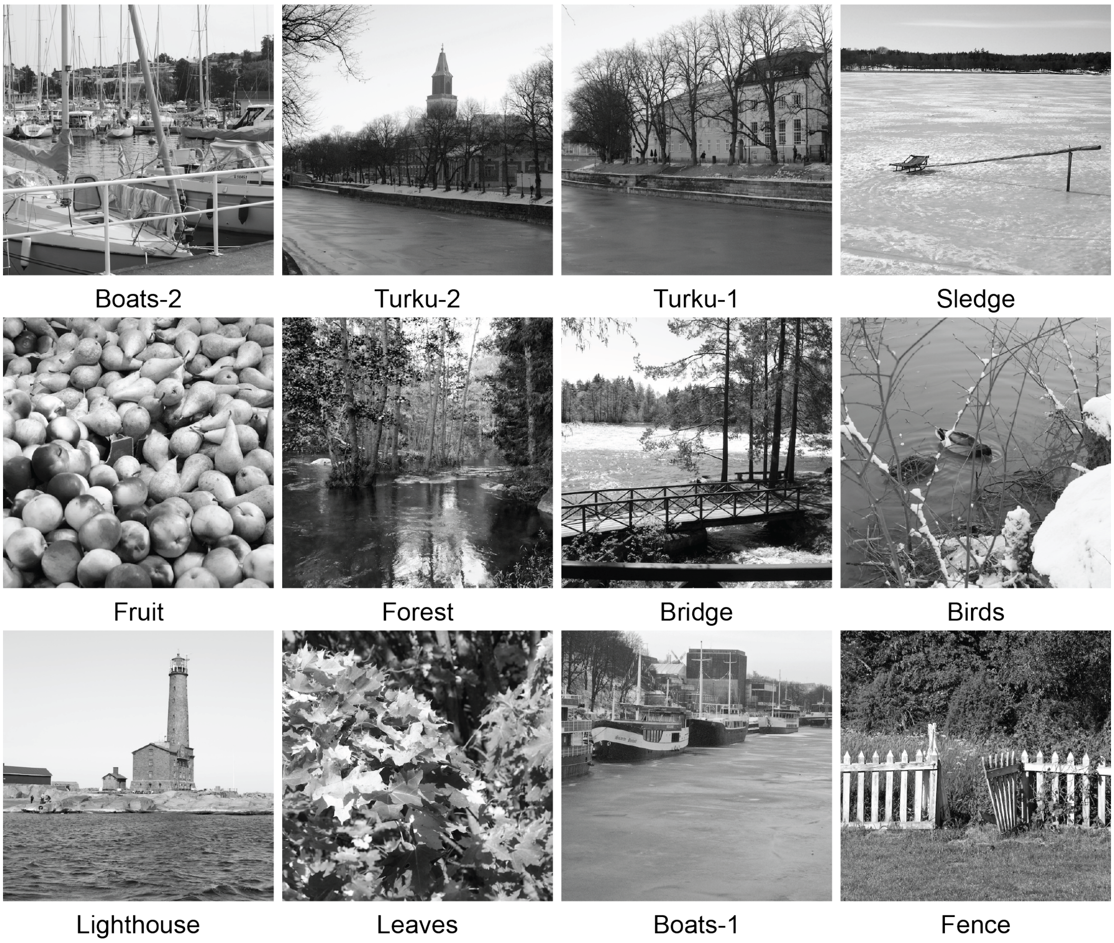

Supplementary Figure 3: The original photographs that were used to create the simulation dataset are shown. The complete simulation dataset consists of Gaussian blurred versions, radii 0-2, of each image. The pictures were taken by S.K.

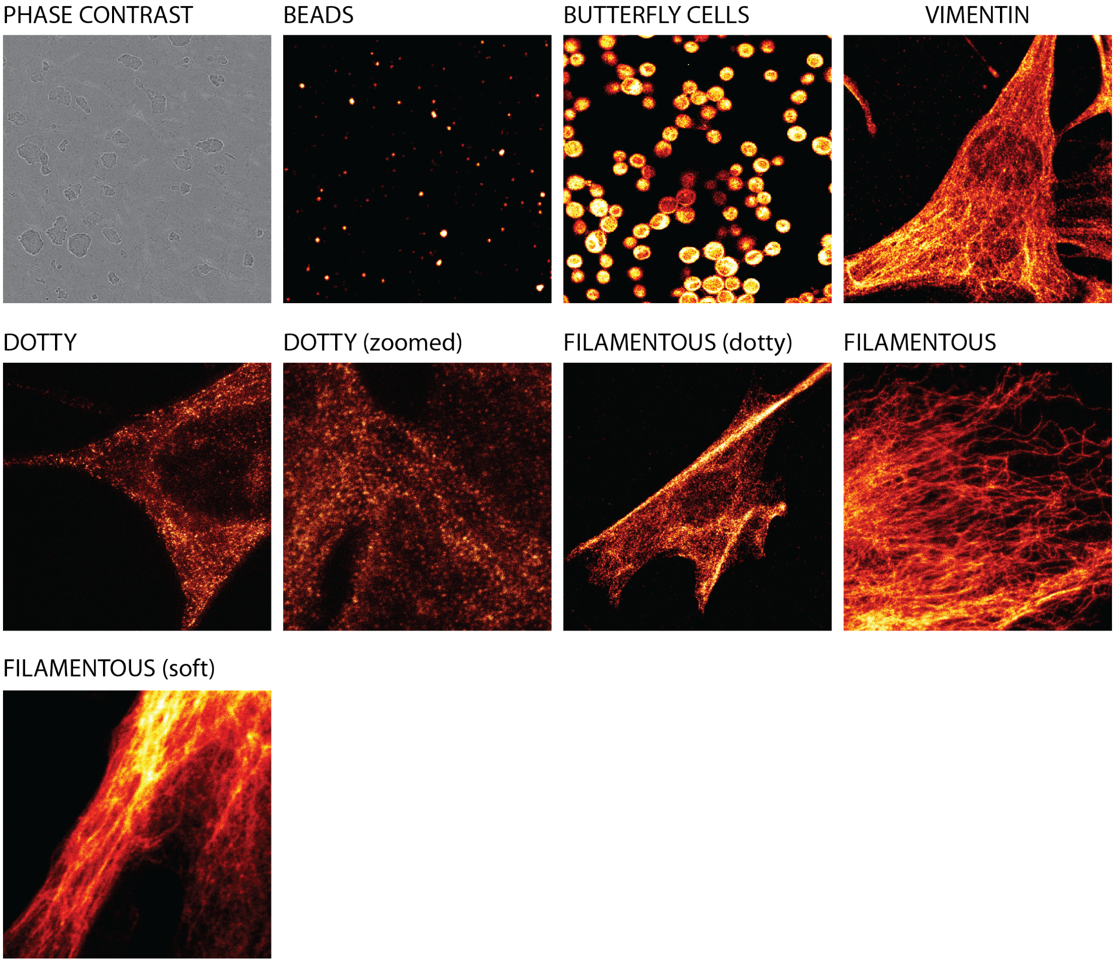

Supplementary Figure 4: The microscope images that were used in the power spectral plots, as well as to create the autofocus simulation datasets are shown. The naming corresponds to that used in the main article.

Supplementary Note 1: A pseudocode description of the *PyImageQualitRanking* software

```
"""
A simplified pseudocode representation of the PyImageQualityRanking
functionality.
"""
options = GetCommandLineOptions(arguments)

# Measure extraction
# In the default directory mode all the files within a given input
# directory are analyzed. The directory is given as a command line
# measure.
if options.directory == True:
        # The results are saved into a CSV data file. The data
        # contains the image filenames as well as all the measure
        # values.
        dataFile = NewCsvFile(options.outputPath)
        dataFile.WriteHeader("Filename", "Entropy", "SpectralSTD" ...)

        for image in fileList(options.path):
                # Only images are considered
                if not IsImageFile(image):
                        continue
                imageFile = OpenImage(image)

                # The program extracts a number of measures in
                # spatial and spectral domains.
                # All the measures are calculated
                # here and then they are saved as a new row to the
                # data file.
                spatialParams = GetSpatialMeasures(imageFile)
                spectralParams = GetSpectralMeasures(imageFile)
                dataFile.writeRow([spatialParams, spectralParams])

        dataFile.Save()

# Measure calculations
# Here the above produced data file is processed using functions in
# the Pandas library. All the measures are normalized to the
# highest measure value within the dataset. In addition certain
# new measures, for example CV or invSTD (1 - spectral
# domain STD) are calculated here.
if options.analyze == True:
        dataFile = ReadCsv(options.outputPath)

        newValues = analyzeData(dataFile)
        dataFile.Append(newValues)
        dataFile.Save()

# Measure ranking
# The sorting option enables the image ranking. The sorting is
# separate from the analysis functionality, because often times one
# would like to sort the data several times by a different measure
# type. Once the data file has been created the sorting can be run
# as many times as one desires.
if options.sort == True:
        if dataFile is None:
                dataFile = ReadCsv(options.outputPath)

        # Sort by measure value
        dataFile = SortData(dataFile, options.sortingMeasure)
        # The changes are always saved to enable direct observation of
        # the results in the CSV file, as well as to make it possible
        # to use the ranking results for selecting or discarding
        # certain images
        dataFile.Save()
```

```
# Show a chosen number of images from the top and the bottom
# of the new ranking.
bestImages = dataFile.GetBest(options.numberToPlot)
worstImages = dataFile.GetWorst(options.numberToPlot)
plot(bestImages)
plot(worstImages)
```
